# Generalized Entropies, Variance and Applications

**DOI:** 10.3390/e22060709

**Published:** 2020-06-26

**Authors:** Abdolsaeed Toomaj, Antonio Di Crescenzo

**Affiliations:** 1 Department of Mathematics and Statistics, Faculty of Basic Sciences and Engineering, Gonbad Kavous University, Basirat Blvd., Shahid Fallahi Street, Gonbad Kavous 4971799151, Golestan Province, Iran; ab.toomaj@gonbad.ac.ir; 2 Dipartimento di Matematica, Università degli Studi di Salerno, Via Giovanni Paolo II n. 132, I-84084 Fisciano (SA), Italy

**Keywords:** generalized cumulative entropy, generalized cumulative residual entropy, variance, mean residual life, stochastic orders, 60E05, 62B10, 62N05, 94A17

## Abstract

The generalized cumulative residual entropy is a recently defined dispersion measure. In this paper, we obtain some further results for such a measure, in relation to the generalized cumulative residual entropy and the variance of random lifetimes. We show that it has an intimate connection with the non-homogeneous Poisson process. We also get new expressions, bounds and stochastic comparisons involving such measures. Moreover, the dynamic version of the mentioned notions is studied through the residual lifetimes and suitable aging notions. In this framework we achieve some findings of interest in reliability theory, such as a characterization for the exponential distribution, various results on *k*-out-of-*n* systems, and a connection to the excess wealth order. We also obtain similar results for the generalized cumulative entropy, which is a dual measure to the generalized cumulative residual entropy.

## 1. Introduction and Background

The notions of uncertainty and information are relative and involve comparisons of distributions in terms of a probabilistic point of view. In information theory a relevant role is played by the concept of entropy; see Shannon [1]. We recall that an absolutely continuous nonnegative random variable *X* with probability density function (pdf) f, the (differential) entropy of *X* is defined as

(1) $$H\left(X\right)=-E\left[\log f\left(X\right)\right]=-\int_{0}^{\infty }f\left(x\right)\log f\left(x\right)\;dx,$$

where E(·) denotes expectation and “log” is the natural logarithm, with the convention 0log0=0.

Despite the many advantages, the Shannon entropy has some limitations; see, e.g., Rao et al. [2] and Schroeder [3]. We recall the following concerns about the differential entropy. (i) H(X) cannot be used when *X* is a mixed random variable. (ii) The differential entropy of an absolutely continuous random variable may take any value on the extended real line, wheres in the discrete case the entropy is always positive. (iii) H(X) is inconsistent, in the sense that for a uniform distribution it may be zero, or negative, or positive. (iv) The differential entropy is decreased by conditioning. Moreover, the vanishing of the conditional differential entropy of *X* given *Y* does not imply that *X* is a function of *Y*, whereas in the discrete case the conditional entropy of *X* given *Y* is equal to zero if and only if *X* is a function of *Y*. (v) In general, the entropy of the empirical distribution does not provide a proper approximation of the differential entropy of an absolutely continuous random variable. (vi) The differential entropy, as the Shannon entropy, is location independent. To overcome these drawbacks, various new alternative measures of uncertainty have been proposed in the recent literature. Here we recall some basic concepts which are involved in their definitions.

Let *X* be a random variable with support (0,∞), having cumulative distribution function (cdf) F(x)=P(X≤x) and survival function $\bar{F}\left(x\right)=1-F\left(x\right)$. As customary, we denote by

$$\Lambda \left(x\right)=-\log\bar{F}\left(x\right)=\int_{0}^{x}\lambda \left(t\right)\;dt,\;x>0$$

the cumulative hazard function and by

(2) $$\lambda \left(t\right)=\frac{d}{dt}\Lambda \left(t\right)=\frac{f(t)}{\bar{F}\left(t\right)},\;t>0$$

the hazard rate function of *X*. Let *X* describe the lifetime of a system. Under the condition that the system has survived up to age t>0, the survival function and the cumulative hazard function of the residual lifetime $X_{t}:=\left[X-t\;|\;X>t\right]$, t>0, are respectively given by

(3) $${\bar{F}}_{t}\left(x\right)=\frac{\bar{F}\left(x+t\right)}{\bar{F}\left(t\right)},\;\Lambda_{t}\left(x\right)=-\log{\bar{F}}_{t}\left(x\right)=\int_{t}^{t+x}\lambda \left(\tau \right)\;d\tau ,\;x\geq 0,$$

where [X|B] refers to a random variable having the same distribution of *X* conditioned on B. The mean residual lifetime (MRL) function of *X* is defined as

(4) $$m\left(t\right)=E\left[X_{t}\right]=E\left[X-t\;|\;X>t\right]=\frac{1}{\bar{F}\left(t\right)}\int_{t}^{\infty }\bar{F}\left(x\right)\;dx,\;t>0.$$

Similarly, one can also introduce the reversed hazard rate function of *X*, given by τ(t)=f(t)/F(t), t>0, and the cumulative reversed hazard function

(5) $$T\left(x\right)=-\log F\left(x\right)=\int_{x}^{\infty }\tau \left(t\right)\;dt,\;x>0.$$

Under the condition that the system has been found failed at age t>0, the inactivity time is defined as $X_{\left[t\right]}=\left[t-X|X\leq t\right]$, t>0. The mean inactivity time (MIT) function of *X* is

(6) $$\tilde{\mu }\left(t\right)=E\left[X_{\left[t\right]}\right]=E\left[t-X|X\leq t\right]=\frac{1}{F(t)}\int_{0}^{t}F\left(x\right)\;dx,\;t>0.$$

It is also known as the mean past lifetime and the mean waiting time function of *X*.

Let us now recall various useful information measures that have been defined recently in terms of the functions reported above. For clarity and brevity, we report them in Table 1. The cumulative residual entropy (CRE) has been proposed and studied by Rao et al. [2] and Rao [4] as an alternative measure of uncertainty; see case (i) of Table 1. This measure is obtained by replacing the pdf with the survival function in (1). The CRE can be expressed as (see, e.g., Mitra [5] and Asadi and Zohrevand [6])

(7) $$E\left(X\right)=E\left[m\left(X\right)\right]=\int_{0}^{\infty }f\left(x\right)m\left(x\right)\;dx.$$

The CRE has been used by Leser [7] in order to measure the elasticity of life expectancy with respect to a proportional change of mortality in life tables. Recently, it has been extended in Psarrakos and Navarro [8] to the generalized cumulative residual entropy (GCRE) of X, for $n\in N_{0}\equiv N\cup \left\{0\right\}=\left\{0,1,2,\ldots \right\}$, as given in (iii) of Table 1. It is worth noting that the GCRE is a dispersion measure related to the (upper) record values of a sequence of independent and identically distributed random variables and to the relevation transform. We recall that the relevation transform of *X*, denoted by $X(X^{\prime })$, is defined through the survival function

$$P\left[X\left(X^{\prime }\right)>x\right]=\bar{F}\left(x\right)\left[1-\Lambda \left(x\right)\right],\;x\geq 0,$$

where $X^{\prime }$ is an independent copy of the lifetime *X*. Specifically, the GCRE refers to a relevation process with underlying distribution function F(x). This process describes the occurrence of failures of items, where a failed item is replaced (or repaired) by another item with the same age. Hence, after denoting by $X_{n}$ the lifetime of the *n*th items, $X_{1}$ has distribution function F(x). Moreover, $X_{n+1}$ conditional on $X_{n}=t$ has the same distribution of $\left[X_{n}-t\;|\;X_{n}>t\right]$, that is, the residual lifetime of $X_{n}$, for all n∈N (see Psarrakos and Navarro [8] and Kapodistria and Psarrakos [9] for details). According to the above notation, we note that the GCRE satisfies the following probabilistic relation (see Corollary 4.1 of [9], and Equation (11) below):

$$E_{n}\left(X\right)=E\left[X_{n+1}\right]-E\left[X_{n}\right],\;n\in N_{0}.$$

Hence, the GCRE provides a measure on the effectiveness of a relevation process, in the sense of the mean. Moreover, it is appropriate to describe useful information on stochastic models connected with the non-homogeneous Poisson process, shock models, preventive maintenance, repairable systems, and standby systems.

For n=0, one has $E_{0}\left(X\right)=E\left(X\right),$ whereas for n=1, the GCRE identifies with the CRE (see Table 1). In order to capture the effect of the age t>0 of a system on the information of the residual distribution, according to Equation (8) of Psarrakos and Navarro [8], the dynamic version of GCRE, denoted DGCRE for short, is defined by substituting $\bar{F}\left(x\right)$ with ${\bar{F}}_{t}\left(x\right)$ in the definition (see cases (iii) and (v) of Table 1). It is evident that $E_{n}\left(X;0\right)=E_{n}\left(X\right)$. Moreover, for n=0 we have

(8) $$E_{0}\left(X;t\right)=m\left(t\right),$$

i.e., the mean residual life function (4). Some properties of $E_{1}\left(X\right)\equiv E\left(X\right)$ and its dynamic version are widely discussed in Asadi and Zohrevand [6] and Navarro et al. [10], among others. An application of the CRE in reliability engineering systems is given by Toomaj et al. [11]. Several properties of the GCRE and the DGCRE, such as bounds, stochastic ordering, aging classes properties, relationships with other entropy concepts, and characterization results, were investigated by Navarro and Psarrakos [12], Psarrakos and Navarro [8], and Psarrakos and Toomaj [13]. Specifically, in the latter paper the GCRE was studied as a risk measure, compared to the standard deviation and the right-tail risk measure, the latter being introduced by Wang [14].

Various dual measures can be introduced by analogy, as can be seen on the right-hand-side of Table 1, by replacing $\bar{F}\left(x\right)$ with F(x), and Λ(x) with T(x). Indeed, Di Crescenzo and Longobardi [15] defined the cumulative entropy (CE) as an alternative measure of uncertainty for the inactivity time by replacing the survival function with the distribution function; see (ii) of Table 1. We also recall that $\mathrm{CE}\left(X\right)=E[\tilde{\mu }\left(X\right)]$. A suitable extension of the CE, in analogy with the GCRE, was defined by Kayal [16] as the generalized cumulative entropy (GCE); see case (iv) of Table 1. Furthermore, Kayal [16] considered the corresponding dynamic version, named dynamic generalized cumulative entropy (DGCE), as given in case (vi) of Table 1. Note that $\lim_{t\to \infty }{\mathrm{CE}}_{n}\left(X;t\right)={\mathrm{CE}}_{n}\left(X\right)$, which coincides with the GCE of *X*. Various results on GCE and DGCE, related to bounds, stochastic ordering, aging classes properties, relationships with other entropy concepts, and characterizations, were obtained by Kayal [16] and Di Crescenzo and Toomaj [17].

The aim of the present paper is to investigate further results on GCRE and GCE. Specifically, we first illustrate an intimate connection between the GCRE and the non-homogeneous Poisson process. Moreover, we perform various stochastic comparisons, and provide various inequalities, including an upper bound for the GCRE in terms of the standard deviation. Some of our results are based on the fact that the GCRE is related to the record values of a sequence of independent and identically distributed (i.i.d.) random variables and the relevation transform, and that its dual, i.e., the GCE, is related to the lower record values and the reversed relevation transform. We recall that, in analogy with the relevation transform, the reversed relevation transform of *X*, denoted by $X[X^{\prime }]$, is defined through the distribution function

$$P\left[X\left[X^{\prime }\right]\leq x\right]=F\left(x\right)\left[1+T\left(x\right)\right],\;x>0,$$

with $X^{\prime }$ an independent copy of the lifetime *X*.

In this paper we also focus on certain connections with the excess wealth order, based on the result that the GCRE can be expressed in terms of the excess wealth transform. Our investigation also deals with classical coherent systems of interest in reliability theory, i.e., *k*-out-of-*n* systems, by focusing on monotonicity properties and comparison results for their residual variance and GCRE.

The rest of this paper is organized as follows: In Section 2 we investigate a connection of the GCRE with the non-homogeneous Poisson processes (NHPP). Further, an expression for the variance of a random variable based the its mean residual lifetime is obtained and the connection of the GCRE with the standard deviation is considered. The connection of the GCRE with the excess wealth order is obtained in Section 3. Dynamic properties of GCRE and of the residual variance are given in Section 4. A characterization of the exponential distribution in terms of the variance residual life function and the dynamic GCE is also provided in Section 4. In Section 5, we discuss applications of the dynamic GCRE and of the residual variance in reliability, also with reference to *k*-out-of-*n* systems. In Section 6, similar results for the GCE are studied, whereas the dynamic GCE and inactivity variance are investigated in Section 7. Finally, Section 8 concludes the paper with some closing remarks.

For simplicity, in the rest of the paper we write $\phi^{n}\left(x\right)$ instead of ${\left[\phi \left(x\right)\right]}^{n}$ for any given function ϕ. Moreover, $\phi^{\prime }$ denotes the derivative of ϕ. Note that the terms increasing and decreasing are used in a non-strict sense.

## 2. Connection of Variance and GCRE

An intimate connection of the GCRE with the expected interepochs (or successive times) of a non-homogeneous Poisson process and the relevation transform has been pointed out by Psarrakos and Navarro [8]. These notions arise naturally in preventive maintenance, repairable systems, cold standby systems, and shock models. In fact, such connection shows that the known results on the GCRE can be used in various applied contexts thanks to the large usefulness of the Poisson process. In particular, there is an intimate connection among non-homogeneous Poisson processes (NHPPs), record values, and minimal repair processes. For an excellent review, we refer the readers to, e.g., Gupta and Kirmani [18] and Kirmani and Gupta [19].

Let *X* be an absolutely continuous nonnegative random variable with pdf f(x) and survival function $\bar{F}\left(x\right)$. Assume that $0\equiv X_{0}\leq X_{1}\leq X_{2}\leq \ldots$ denote the epoch times of a NHPP with intensity function (2), where $X_{1}$ has the same distribution as *X*. In this case, $X_{n+1}-X_{n},$$n\in N_{0},$ describes the duration of the interepoch intervals or the interoccurrence times. Denoting by ${\bar{F}}_{n+1}\left(x\right)$ the survival function of $X_{n+1},$$n\in N_{0},$ it follows that (see Baxter [20])

(9) $${\bar{F}}_{n+1}\left(x\right)=\bar{F}\left(x\right)\;\sum_{k=0}^{n}\frac{\Lambda^{k}\left(x\right)}{k!},\;x\geq 0,$$

so that the pdf of $X_{n+1}$ is given by

(10) $$f_{n+1}\left(x\right)=f\left(x\right)\frac{\Lambda^{n}\left(x\right)}{n!},\;x\geq 0.$$

Recalling the definition of GCRE given in case (iii) of Table 1, from (9) we obtain

(11) $$\begin{array}{ccc} E_{n}\left(X\right)\;\;\;\; & = & \;\;\;\;E\left[X_{n+1}-X_{n}\right]=\int_{0}^{\infty }\left[{\bar{F}}_{n+1}\left(x\right)-{\bar{F}}_{n}\left(x\right)\right]dx=\int_{0}^{\infty }\frac{\Lambda^{n}\left(x\right)}{n!}\bar{F}\left(x\right)\;dx,\;n\in N_{0}. \end{array}$$

Thus, for $n\in N_{0},$ the GCRE of order *n* corresponds to the expected interepoch interval in a non-homogeneous Poisson process, with $E_{0}\left(X\right)=E\left(X\right)$.

As mentioned by Psarrakos and Toomaj [13], there is a close relationship between the GCRE and the standard deviation σ(X). Indeed, $E_{n}\left(X\right),$n∈N can be used for measuring the closeness of *X* to a degenerate distribution; i.e., it is a dispersion measure.

**Proposition** **1.** *Let Y=ϕ(X), where ϕ:(0,∞)→(0,∞) is a strictly increasing function such that $\phi^{\prime }\left(u\right)\geq 1$ for all u>0. Then, $E_{n}\left(X\right)\leq E_{n}\left(Y\right)$ for all n∈N.*

**Proof.**  From (iii) of Table 1, denoting by $\phi^{-1}$ the inverse of ϕ, one has

$$\begin{array}{ccc} E_{n}\left(Y\right)\;\;\;\; & = & \frac{1}{n!}\int_{0}^{\infty }{\bar{F}}_{Y}\left(x\right){\left[-\log{\bar{F}}_{Y}\left(x\right)\right]}^{n}\;dx\\ \; & = & \frac{1}{n!}\int_{0}^{\infty }{\bar{F}}_{X}\left(\phi^{-1}\left(x\right)\right){\left[-\log{\bar{F}}_{X}\left(\phi^{-1}\left(x\right)\right)\right]}^{n}dx\\ \; & = & \frac{1}{n!}\int_{0}^{\infty }\phi^{\prime }\left(u\right){\bar{F}}_{X}\left(u\right){\left[-\log{\bar{F}}_{X}\left(u\right)\right]}^{n}du\\ \; & = & E_{n}\left(X\right)+\frac{1}{n!}\int_{0}^{\infty }\left[\phi^{\prime }\left(u\right)-1\right]{\bar{F}}_{X}\left(u\right){\left[-\log{\bar{F}}_{X}\left(u\right)\right]}^{n}\;du. \end{array}$$

Therefore, the proof immediately follows. □

This result is similar to that shown in Theorem 1 of Ebrahimi et al. [21] for the differential entropy. It should be noted that one advantage of the GCRE is that it exists for heavy tailed distributions with mean E(X)<∞ and $E(X^{2})=\infty$, while in this case σ(X) is not finite; see Psarrakos and Toomaj [13] and Yang [22]. Similar results also hold for the generalized entropy.

Hereafter, we provide a connection between the variance and the generalized cumulative residual entropy. First, we obtain an expression for the variance, $\sigma^{2}\left(X\right)$, in terms of the mean residual life function. It is based on the results in Hall and Wellner [23] (see also, Fernandez-Ponce et al. [24]).

**Theorem** **1.** *Let X be an absolutely continuous nonnegative random variable with pdf f, distribution function F, and mean residual life function m(x), where the second moment $E(X^{2})$ is finite. Then*

(12) $$\sigma^{2}\left(X\right)=E\left[m^{2}\left(X\right)\right].$$

Hereafter we show that Theorem 1 does not hold for a discrete random variable.

**Remark** **1.** Let *X* be an integer-valued random variable with probability mass function p(j)=P(X=j) and survival function $\bar{P}\left(k\right)=P\left(X\geq k\right)=\sum_{i=k}^{\infty }p\left(i\right),$ respectively. The discrete mean residual life m(k) is defined as (see Roy and Gupta [25])

(13) $$m\left(k\right)=E\left[X-k|X>k\right]=\frac{1}{\bar{P}\left(k+1\right)}\sum_{j=k+1}^{\infty }\bar{P}\left(j\right),\;k\in N_{0}.$$

For example, we consider a three-point discrete distribution with the probability mass function p(0)=1/2,p(1)=1/4 and p(2)=1/4. Then from (13), we obtain m(0)=3/2 and m(1)=1 while m(2)=0. It is obvious that $\sigma^{2}\left(X\right)=11/16.$ However, we get

$$E\left[m^{2}\left(X\right)\right]=\sum_{j=0}^{2}m^{2}\left(j\right)p\left(j\right)=\frac{11}{8}\neq \sigma^{2}\left(X\right).$$

Applying Theorem 1, one can get variance inequalities under suitable sufficient conditions. First, we recall that *X* is said to have increasing (decreasing) mean residual life, IMRL (DMRL) for short, if m(t) is increasing (decreasing) in t>0.

Moreover, for absolutely continuous nonnegative random variables *X* and *Y* with CDFs *F* and *G*, PDFs *f* and g, and mean residual lifetime functions $m_{X}\left(t\right)$ and $m_{Y}\left(t\right),$ respectively, we say that *X* is smaller than *Y* in the

- Usual stochastic order, denoted by $X\leq_{\mathrm{st}}Y,$ if $\bar{F}\left(t\right)\leq \bar{G}\left(t\right),\;\forall \;t\in R;$
- Mean residual lifetime order, denoted by $X\leq_{\mathrm{mrl}}Y,$ if $m_{X}\left(t\right)\leq m_{Y}\left(t\right)$ for all t>0.

For further information and properties about these concepts and on the stochastic orders that will be used in this paper (i.e., $\leq_{hr}$, $\leq_{rhr}$, $\leq_{ew}$, $\leq_{disp}$), we refer the reader to Shaked and Shanthikumar [26]. In view of the following theorem, we recall that neither of the orders $\leq_{st}$ and $\leq_{mrl}$ implies the other (see Section 2.A.2 of [26]).

**Theorem** **2.** *Let X and Y be nonnegative random variables with mean residual life functions $m_{X}\left(x\right)$ and $m_{Y}\left(x\right),$ respectively. Then*

 **(i)**  *Let $X\leq_{st}Y$ and $X\leq_{mrl}Y$. If either X or Y is IMRL; then $\sigma^{2}\left(X\right)\leq \sigma^{2}\left(Y\right).$*
 **(ii)**  *Let $X\geq_{st}Y$ and $X\geq_{mrl}Y$. If either X or Y is DMRL; then $\sigma^{2}\left(X\right)\geq \sigma^{2}\left(Y\right).$*

**Proof.**  Let *Y* be IMRL. From (12), we get

$$\begin{array}{c} \sigma^{2}\left(X\right)=E\left[m_{X}^{2}\left(X\right)\right]\leq E\left[m_{Y}^{2}\left(X\right)\right]\leq E\left[m_{Y}^{2}\left(Y\right)\right]=\sigma^{2}\left(Y\right). \end{array}$$

The first inequality is obtained from the assumption $X\leq_{mrl}Y$ while the last inequality is obtained by the fact that $X\leq_{st}Y$ implies that E(ψ(X))≤E(ψ(Y)) for all increasing functions ψ(·). Now, let *X* be IMRL. Then, we similarly have

$$\begin{array}{c} \sigma^{2}\left(X\right)=E\left[m_{X}^{2}\left(X\right)\right]\leq E\left[m_{X}^{2}\left(Y\right)\right]\leq E\left[m_{Y}^{2}\left(Y\right)\right]=\sigma^{2}\left(Y\right), \end{array}$$

and hence the result stated in (i) follows. The proof of (ii) is similar. □

**Example** **1.** Let X∼GP(a,b) have generalized Pareto distribution, with survival function

$$\bar{F}\left(x\right)={\left(\frac{b}{ax+b}\right)}^{\frac{1}{a}+1},\;x\geq 0,\;\mathrm{with}\;\;x<-\frac{b}{a}\;\;\mathrm{if}\;a<0,$$

for a>−1, a≠0, b>0 (see Arriaza et al. [27] for a recent characterization of the generalized Pareto distribution). It is well known that *X* has linear mean residual life function m(x)=ax+b, x≥0, ax+b>0. Clearly, *X* is DMRL if −1<a<0, is exponential if a→0, and is IMRL if a>0. Assume that Y∼GP(c,d), with analogous constraints on the parameters. It is clear that if a=c and b<d, then $X\leq_{st}Y$ and $X\leq_{mrl}Y$. If, in addition, a=c>0, then both *X* and *Y* are IMRL. Indeed, in this case the result stated in point (i) of Theorem 2 is confirmed, being $\sigma^{2}\left(X\right)=\frac{1+a}{1-a}\;b^{2}<\frac{1+c}{1-c}\;d^{2}=\sigma^{2}\left(Y\right)$ for a=c∈(−1,1) and 0<b<d.

In the sequel, we give an upper bound for the generalized CRE in terms of the standard deviation. First, as extension of Theorem 2.1 of Asadi and Zohrevand [6] we provide an expression for the GCRE in terms of the mean residual life function.

**Theorem** **3.** *Let X be an absolutely continuous nonnegative random variable with survival function $\bar{F}\left(x\right)$ and mean residual life function m(x), and let $X_{n}$ be the nth epoch time of a NHPP with intensity function (2). Then, for all n∈N*

(14) $$E_{n}\left(X\right)=E\left[m\left(X_{n}\right)\right].$$

The identity (14) is a special case of Equation (34), which is proved in Section 4 below. As application of the representations (12) and (14), consider the following results.

**Example** **2.** (i) Assume that *X* is exponentially distributed with mean μ. Then, it is known that the MRL function of *X* is m(t)=μ,t>0. Hence, we have $\sigma^{2}\left(X\right)=\mu^{2},$ and $E_{n}\left(X\right)=\mu ,$ for any n∈N due to (14).

(ii) If *X* is distributed uniformly on (0,b),b>0, then m(t)=(b−t)/2,0≤t≤b. From this, it can be shown that $\sigma^{2}\left(X\right)=E\left({\left(b-X\right)}^{2}/4\right)=b^{2}/12.$ Moreover, for any n∈N we have

$$E_{n}\left(X\right)=\frac{b}{2}-\frac{1}{2}E\left[X_{n}\right]=\frac{b}{2^{n+1}},$$

where the last equality is obtained by noting that $E\left[X_{n}\right]=b-b/2^{n}.$

(iii) Let *X* have a Pareto (Lomax) distribution with parameters α>1 and β>0, with survival function and mean residual life given respectively by

$$\bar{F}\left(x\right)=\frac{\beta^{\alpha }}{{\left(x+\beta \right)}^{\alpha }},\;x\geq 0,\;m\left(t\right)=\frac{1}{\alpha -1}\left(t+\beta \right),\;t\geq 0.$$

Hence, we get

$$\sigma^{2}\left(X\right)=\frac{1}{{\left(\alpha -1\right)}^{2}}E\left[{\left(X+\beta \right)}^{2}\right]=\frac{\alpha \beta^{2}}{{\left(\alpha -1\right)}^{2}\left(\alpha -2\right)}.$$

Furthermore, it holds that

$$E_{n}\left(X\right)=\frac{1}{\alpha -1}\left(E[X_{n}]+\beta \right)=\frac{\beta \alpha^{n}}{{\left(\alpha -1\right)}^{n+1}},$$

where the last equality is obtained by $E\left[X_{n}\right]=\beta \alpha^{n}/{\left(\alpha -1\right)}^{n}-\beta$, for n∈N.

**Remark** **2.** A class of distributions of interest in maintenance policies and shock models is the class of new better (worse) than used in expectation (NBUE) (NWUE) distributions. A random lifetime *X* is said to have a new better (worse) than used in expectation (NBUE) (NWUE) distribution if m(t)≤(≥)m(0)=μ for all t>0. For a greater detail on these concepts, we refer the reader to, e.g., Barlow and Proschan [28]. If *X* is NBUE (NWUE), then under the assumptions of Theorem 3 we have

(15) $$E_{n}\left(X\right)\leq \left(\geq \right)\;\mu .$$

Moreover, from (12) one can see that $\sigma^{2}\left(X\right)\leq \left(\geq \right)\;\mu^{2}$, or equivalently

(16) σ(X)≤(≥)μ.

The upper (lower) bounds in (15) and (16) show that the expected interepoch intervals (or the amount of uncertainty of a random variable *X* based on the GCRE) and the standard deviation are less (greater) than the expected value of *X* if it is NBUE (NWUE).

Thanks to (14), an iterative formula for the GCRE is proved in the following theorem.

**Theorem** **4.** *Under the assumption of Theorem 3, for all n∈N we have*

(17) $$E_{n}\left(X\right)=E_{n-1}\left(X\right)+\frac{1}{(n-1)!}\;E\left[h_{n}\left(X\right)\right],$$

*where*

$$h_{n}\left(t\right):=\int_{0}^{t}m^{\prime }\left(x\right)\Lambda^{n-1}\left(x\right)\;dx.$$

**Proof.**  By virtue of (14) and $m\left(x\right)\lambda \left(x\right)=1+m^{\prime }\left(x\right)$, it holds that

$$E_{n}\left(X\right)=\int_{0}^{\infty }m\left(x\right)\lambda \left(x\right)\bar{F}\left(x\right)\frac{\Lambda^{n-1}\left(x\right)}{(n-1)!}\;dx=E_{n-1}\left(X\right)+\int_{0}^{\infty }m^{\prime }\left(x\right)\bar{F}\left(x\right)\frac{\Lambda^{n-1}\left(x\right)}{(n-1)!}\;dx,$$

for all n∈N. Using Fubini’s theorem, we have

$$\begin{array}{ccc} \int_{0}^{\infty }m^{\prime }\left(x\right)\bar{F}\left(x\right)\Lambda^{n-1}\left(x\right)\;dx & = & \int_{0}^{\infty }m^{\prime }\left(x\right)\int_{x}^{\infty }f\left(u\right)\Lambda^{n-1}\left(x\right)\;du\;dx\\ \; & = & \int_{0}^{\infty }f\left(u\right)\int_{0}^{u}m^{\prime }\left(x\right)\Lambda^{n-1}\left(x\right)\;dx\;du, \end{array}$$

this giving the desired result. □

Hereafter we provide an alternative iterative formula for the GCRE.

**Theorem** **5.** *Under the assumption of Theorem 3, for all n∈N, we have*

(18) $$E_{n}\left(X\right)=E_{n-1}\left(X\right)\left\{1+E[m^{\prime }\left(Z\right)]\right\},$$

*where Z is an absolutely continuous nonnegative random variable having pdf*

$$f_{Z}\left(x\right)=\frac{\bar{F}\left(x\right)}{E_{n-1}\left(X\right)}\;\frac{\Lambda^{n-1}\left(x\right)}{(n-1)!},\;x>0.$$

**Proof.**  The result follows from the probabilistic mean value theorem (cf. Theorem 4.1 of [29]) and making use of Equations (11) and (14). □

**Remark** **3.** *It is worth mentioning that if X is IMRL (DMRL), since $m^{\prime }\left(x\right)\geq \left(\leq \right)\;0,$ then as an immediate consequence of (17) or (18), we have*

$$E_{n}\left(X\right)\geq \left(\leq \right)\;E_{n-1}\left(X\right)\;\mathrm{for}\;\mathrm{all}\;n\in N.$$

Hereafter, we obtain an upper bound for the GCRE of *X* in terms of its standard deviation, thereby providing a suitable relation between two concepts of dispersion measure.

**Theorem** **6.** *Let X be an absolutely continuous nonnegative random variable with standard deviation σ(X) and GCRE function $E_{n}\left(X\right).$ Then, for all n∈N,*

(19) $$E_{n}\left(X\right)\leq \frac{\sqrt{[2(n-1)]!}}{(n-1)!}\;\sigma \left(X\right),$$

**Proof.**  By the Cauchy–Schwarz inequality, for all n∈N we obtain

$$\begin{array}{c} {\left[\int_{0}^{\infty }m\left(x\right)\Lambda^{n-1}\left(x\right)f\left(x\right)\;dx\right]}^{2}\leq \left(\int_{0}^{\infty }m^{2}\left(x\right)f\left(x\right)\;dx\right)\left(\int_{0}^{\infty }\Lambda^{2n-2}\left(x\right)f\left(x\right)\;dx\right). \end{array}$$

On the other hand, it holds that

(20) $$\int_{0}^{\infty }\Lambda^{2n-2}\left(x\right)f\left(x\right)\;dx=\Gamma \left(2n-1\right)=\left[2\left(n-1\right)\right]!.$$

Therefore, use of Theorems 1 and 3 completes the proof. □

In the special case n=1, from Theorem 6 one can obtain a close relationship between the standard deviation and CRE; i.e.,

(21) $$e^{CH(X)}\leq E_{1}\left(X\right)\leq \sigma \left(X\right),$$

where, for $C=\exp\{\int_{0}^{1}\log(x\;|\log x|)\;dx\}≅0.2065$, the first inequality is obtained from Rao et al. [2]. It is worth pointing out that the inequalities given in (21) involve three uncertainty measures. Moreover, the related inequality between the differential entropy and the standard deviation is similar to relation (2) of Ebrahimi et al. [21]. They also pointed out that the first inequality in (21) becomes an equality if and only if *X* is normal. Moreover, if *X* is exponential then the second inequality is satisfied as equality. As well known, the entropy is a measure of disparity of the density function f(x) from the uniform distribution. On the other hand, the variance measures an average of distances of outcomes of the probability distribution f(x) from the mean. Additionally, as mentioned by Psarrakos and Toomaj [13] (see also Toomaj et al. [11]), the CRE acts like the standard deviation, i.e., it is a dispersion measure in spite of the similarity shape with the Shannon entropy. Although all of three measures, i.e., entropy, standard deviation, and CRE, are measures of dispersion and uncertainty, the lack of a simple relationship between orderings of a distribution by the three measures derives from their quite substantial and subtle differences. All such measures reflect “concentration”, but their respective metrics for concentration are different.

We remark also that the Stirling formula allows one to obtain the following asymptotic result for the term given in the right-hand-side of (20):

$$\frac{\sqrt{[2(n-1)]!}}{(n-1)!}∼\frac{2^{n-1}}{{\left[\pi \left(n-1\right)\right]}^{1/4}}\;\mathrm{as}\;n\to \infty .$$

For the last theorem of this section, we provide suitable bounds for the GCRE. These are useful in reliability applications, since in a typical situation the only fact known a priori, for example, is that the component has an increasing (decreasing) failure rate due to wear (reverse ageing). We recall that if *X* is an absolutely continuous nonnegative random variable with hazard rate function λ(t), then *X* is said to have an increasing (decreasing) failure rate, say IFR (DFR), if λ(t) is increasing (decreasing) in t>0.

**Theorem** **7.** *Let X be an absolutely continuous nonnegative random variable with hazard rate function λ(t) and GCRE function $E_{n}\left(X\right)$. If X is IFR (DFR), then for all n∈N*

$$E_{n}\left(X\right)\leq \left(\geq \right)\;\frac{1}{n!}\;E\left[X^{n}\lambda^{n-1}\left(X\right)\right].$$

**Proof.**  Since *X* is IFR (DFR), for all x≥0 we have

(22) $$\Lambda \left(x\right)=\int_{0}^{x}\lambda \left(t\right)\;dt\leq \left(\geq \right)\;x\lambda \left(x\right).$$

Hence, recalling that *X* has survival function $\bar{F}\left(t\right)$ and pdf f(x), from (iii) of Table 1 we have

$$n!\;E_{n}\left(X\right)=\int_{0}^{\infty }\bar{F}\left(x\right){\left[\Lambda \left(x\right)\right]}^{n}\;dx\leq \left(\geq \right)\int_{0}^{\infty }x^{n}\lambda^{n}\left(x\right)\bar{F}\left(x\right)\;dx=\int_{0}^{\infty }x^{n}\lambda^{n-1}\left(x\right)f\left(x\right)\;dx,$$

this giving the proof. □

It is not hard to verify that if *X* is IFR, then

(23) $$E\left[X\Lambda^{n-1}\left(X\right)\right]\leq E\left[X^{n}\lambda^{n-1}\left(X\right)\right],\;\mathrm{for}\;\mathrm{all}\;n\in N.$$

We recall that the condition that *X* is IFR implies that *X* is IFRA, i.e., $-\frac{1}{t}\log\bar{F}\left(t\right)$ is increasing in *t*, so that the above relation indicates that the upper bound in Proposition 3.1 of Psarrakos and Toomaj [13] is a sharper bound. On the other hand, if *X* is DFR, then it is also DFRA. In this case, the inequality in (23) is reversed. This shows also that the lower bound in Proposition 3.1 of [13] improves the bound given in Theorem 7 when *X* is DFR.

## 3. Connection with the Excess Wealth Order

One of the most important issues in statistics, probability, actuarial science, risk theory, and other related areas is the concept of variability. The simplest way of comparing the variabilities of two distributions involves the associated standard deviations. However, the comparison of numerical measures is not always sufficiently informative. Several transforms and stochastic orders for comparing their variabilities have been introduced and widely studied (see Shaked and Shanthikumar [26]). One of said orders for the measure of spread is the excess wealth order. For a nonnegative random variable *X* with probability distribution function F, we recall that the quantile function is defined by

$$F^{-1}\left(p\right)=\sup\left\{x\geq 0:F\left(x\right)\leq p\right\},\;\mathrm{for}\;p\in \left(0,1\right).$$

The excess wealth transform (or right spread function) is, for p∈(0,1),

(24) $$W_{X}\left(p\right)=\int_{F^{-1}\left(p\right)}^{\infty }\bar{F}\left(x\right)\;dx=\int_{p}^{1}\left(F^{-1}\left(q\right)-F^{-1}\left(p\right)\right)\;dq.$$

This function is also related to the mean residual life function (4) by the following relation

(25) $$m_{X}\left(F^{-1}\left(p\right)\right)=\frac{W_{X}\left(p\right)}{1-p},\;0<p<1.$$

For an absolutely continuous nonnegative random variable *X* with strictly increasing distribution function *F*, Fernández-Ponce et al. [24] obtained the following representation for the variance of *X* in terms of excess wealth

$$\sigma^{2}\left(X\right)=\int_{0}^{1}{\left[m_{X}\left(F^{-1}\left(p\right)\right)\right]}^{2}\;dp.$$

Analogously, in the following theorem we show that the GCRE can be expressed in terms of the excess wealth transform by means of (14). The proof is straightforward and thus is omitted.

**Theorem** **8.** *For an absolutely continuous nonnegative random variable X, for any n∈N one has*

(26) $$E_{n}\left(X\right)=\frac{1}{(n-1)!}\int_{0}^{1}m_{X}\left(F^{-1}\left(p\right)\right){\left[-\log\left(1-p\right)\right]}^{n-1}\;dp.$$

**Example** **3.** (i) If *X* is uniformly distributed in [0,b], then $m_{X}\left(F^{-1}\left(p\right)\right)=b\left(1-p\right)/2$. Thus, using (26), for any n∈N we obtain

$$E_{n}\left(X\right)=\frac{b}{2(n-1)!}\int_{0}^{1}\left(1-p\right){\left[-\log\left(1-p\right)\right]}^{n-1}\;dp=\frac{b}{2^{n+1}}.$$

(ii) Let us consider the Pareto distribution given in case (iii) of Example 2. It is easily seen that $m_{X}\left(F^{-1}\left(p\right)\right)=\beta {\left(1-p\right)}^{-1/\alpha }/\left(\alpha -1\right)$. Thus, from (26), for any n∈N we have

$$E_{n}\left(X\right)=\frac{\beta }{(\alpha -1)(n-1)!}\int_{0}^{1}{\left(1-p\right)}^{-1/\alpha }{\left[-\log\left(1-p\right)\right]}^{n-1}dp=\frac{\beta \alpha^{n}}{{\left(\alpha -1\right)}^{n+1}}.$$

Let *X* and *Y* be random variables with distribution functions *F* and G, respectively. Then *X* is said to be smaller than *Y* in the excess wealth order, denoted as $X\leq_{ew}Y,$ when $W_{X}\left(p\right)\leq W_{Y}\left(p\right)$ for all p∈(0,1). It is known that $X\leq_{ew}Y$ implies $\sigma^{2}\left(X\right)\leq \sigma^{2}\left(Y\right)$; see Shaked and Shanthikumar [26]. From (26) we thus obtain the following result, whose proof is straightforward.

**Theorem** **9.** *Let X and Y be absolutely continuous nonnegative random variables. If $X\leq_{ew}Y,$ then $E_{n}\left(X\right)\leq E_{n}\left(Y\right),$ for any n∈N.*

Consequently, we have the following implications:

$$X\leq_{disp}Y\;⟹\;X\leq_{ew}Y\;⟹\;E_{n}\left(X\right)\leq E_{n}\left(Y\right),$$

for any n∈N. We recall that *X* is smaller than *Y* in the dispersive order (denoted by $X\leq_{disp}Y$) if $F^{-1}\left(v\right)-F^{-1}\left(u\right)\leq G^{-1}\left(v\right)-G^{-1}\left(u\right)$, ∀0<u≤v<1 (for more details, see, e.g., Shaked and Shanthikumar [26]).

## 4. Results on Dynamic GCRE and Residual Variance

In this section, we extend the previous results from GCRE to the DGCRE, cf. Table 1, as well as to the variance residual life function. The mean residual life function at age *t* has been employed in life lengths studies by various authors; see, e.g., Hollander and Proschan [30], Bhattacharjee [31], Hall and Wellner [23], Gupta [32], and the references therein. Another quantity which has generated interest in recent years is the variance of the residual life function defined by

(27) $$\begin{array}{ccc} \sigma^{2}\left(X_{t}\right) & = & V\mathrm{ar}\left[X-t\;|\;X>t\right]=E\left[{\left(X-t\right)}^{2}|X>t\right]-m^{2}\left(t\right)\\ \; & = & \frac{2}{\bar{F}\left(t\right)}\int_{t}^{\infty }\int_{x}^{\infty }\bar{F}\left(u\right)\;du\;dx-m^{2}\left(t\right),\;t>0. \end{array}$$

It is involved in the formula for $V\mathrm{ar}[{\hat{m}}_{n}\left(t\right)]$ where ${\hat{m}}_{n}\left(t\right)$ is an estimator of the mean residual life function; see Hall and Wellner [23]. Several properties of the variance residual life function are studied in Gupta [32], Gupta et al. [33], and Gupta and Kirmani [18], among others. In this section, we will study some further results of this quantity and the GCRE. Similarly to (12), one can obtain an analogue representation for the variance residual life function in terms of the mean residual life function as follows:

(28) $$\sigma^{2}\left(X_{t}\right)=E\left[m^{2}\left(X\right)\;|\;X>t\right]=\frac{1}{\bar{F}\left(t\right)}\int_{t}^{\infty }m^{2}\left(x\right)f\left(x\right)\;dx,$$

for all t>0. Furthermore, its derivative is expressed as (see also Gupta [32])

(29) $$\frac{d}{dt}\sigma^{2}\left(X_{t}\right)=\lambda \left(t\right)\left[\sigma^{2}\left(X_{t}\right)-m^{2}\left(t\right)\right],\;t>0.$$

If *X* is an absolutely continuous nonnegative random variable, it is shown in Psarrakos and Navarro [8] and Navarro and Psarrakos [12] that, for t>0 and n∈N, the DGCRE satisfies the following equalities:

(30) $$E_{n}^{\prime }\left(X;t\right)=\lambda \left(t\right)\left[E_{n}\left(X;t\right)-E_{n-1}\left(X;t\right)\right],$$

and

(31) $$E_{n}\left(X;t\right)=\frac{1}{\bar{F}\left(t\right)}\int_{t}^{\infty }E_{n-1}\left(X;x\right)\;f\left(x\right)\;dx.$$

Moreover, recalling (3), in analogy with (11) we have:

(32) $$\begin{array}{c} E_{n}\left(X;t\right)=E\left[X_{n+1}-X_{n}\;|\;X>t\right]=\int_{0}^{\infty }{\bar{F}}_{t}\left(x\right)\frac{{\left[\Lambda_{t}\left(x\right)\right]}^{n}}{n!}\;dx,\;n\in N_{0}. \end{array}$$

Hereafter we specify the dynamic version of identity (14). To this aim we point out that

(33) $$f_{n}\left(x\;|\;t\right):=\frac{{\left[\Lambda \left(x\right)-\Lambda \left(t\right)\right]}^{n-1}}{(n-1)!}\frac{f(x)}{\bar{F}\left(t\right)},\;x\geq t\geq 0$$

is the pdf of $\left[X_{n}\;|\;X>t\right]$, n∈N. Recalling the correspondence between the structure of upper record values and the occurrence times of non-homogeneous Poisson processes under minimal repair (see Gupta and Kirmani [18]), we note that $f_{n}\left(x\;|\;t\right)$ can be viewed also as the pdf of the *n*th epoch time of a (delayed) NHPP Poisson process whose intensity function is $\lambda \left(x\right)\;1_{\left\{x\geq t\right\}}$.

**Theorem** **10.** *Under the assumptions of Theorem 1, for all n∈N and t≥0 we have*

(34) $$E_{n}\left(X;t\right)=E\left[m\left(X_{n}\right)\;|\;X>t\right].$$

**Proof.**  Recalling (3), since $\lambda_{t}\left(z\right)=\frac{d}{dz}\Lambda_{t}\left(z\right)=\lambda \left(t+z\right)$, it is not hard to see that for all n∈N one has

$$\int_{0}^{u}\frac{\Lambda_{t}^{n-1}\left(z\right)}{(n-1)!}\lambda_{t}\left(z\right)\;dz=\frac{\Lambda_{t}^{n}\left(u\right)}{n!},\;u,t\geq 0.$$

From the above relation, from the definition given in (iii) of Table 1, and using Fubini’s theorem, we obtain

$$\begin{array}{ccc} E_{n}\left(X;t\right) & = & \int_{0}^{\infty }\frac{\Lambda_{t}^{n}\left(u\right)}{n!}{\bar{F}}_{t}\left(u\right)\;du=\int_{0}^{\infty }{\bar{F}}_{t}\left(u\right)\int_{0}^{u}\frac{\Lambda_{t}^{n-1}\left(z\right)}{(n-1)!}\lambda_{t}\left(z\right)\;dz\;du\\ \; & = & \int_{0}^{\infty }\frac{\Lambda_{t}^{n-1}\left(z\right)}{(n-1)!}\lambda_{t}\left(z\right)\int_{z}^{\infty }{\bar{F}}_{t}\left(u\right)\;du\;dz\\ \; & = & \int_{0}^{\infty }\frac{\Lambda_{t}^{n-1}\left(z\right)}{(n-1)!}\frac{f(z+t)}{\bar{F}\left(z+t\right)}\int_{z}^{\infty }\frac{\bar{F}\left(u+t\right)}{\bar{F}\left(t\right)}\;du\;dz. \end{array}$$

By setting y=u+t and recalling (4), we have

$$\begin{array}{ccc} E_{n}\left(X;t\right) & = & \int_{0}^{\infty }\frac{\Lambda_{t}^{n-1}\left(z\right)}{(n-1)!}\frac{f(z+t)}{\bar{F}\left(t\right)}\int_{z+t}^{\infty }\frac{\bar{F}\left(y\right)}{\bar{F}\left(z+t\right)}\;dy\;dz\\ \; & = & \int_{0}^{\infty }\frac{\Lambda_{t}^{n-1}\left(z\right)}{(n-1)!}\frac{f(z+t)}{\bar{F}\left(t\right)}\;m\left(z+t\right)\;dz\\ \; & = & \int_{t}^{\infty }\frac{{\left[\Lambda \left(x\right)-\Lambda \left(t\right)\right]}^{n-1}}{(n-1)!}\frac{f(x)}{\bar{F}\left(t\right)}\;m\left(x\right)\;dx, \end{array}$$

with x=z+t. Thus, due to (33), we get the result (34). □

Clearly, for t=0, Equation (34) yields identity (14). We remark that the dynamic version of Theorem 6 provides the following upper bound:

$$E_{n}\left(X;t\right)\leq \frac{\sqrt{[2(n-1)]!}}{(n-1)!}\;\sigma \left(X_{t}\right),\;n\in N,\;t\geq 0.$$

In the next theorem, through two suitable expressions we provide different probabilistic meanings for the DGCRE. The first is given in terms of a conditional expectation involving the cumulative hazard function and the hazard rate function. The second involves the conditional covariance of the *n*-th epoch time and the random variable $\Lambda (X_{n})$.

**Theorem** **11.** *For any t≥0 and for all n∈N, it holds that*

**(i)**  $\frac{1}{n}\;E\left[\frac{\Lambda \left(X_{n}\right)-\Lambda \left(t\right)}{\lambda (X_{n})}\;|\;X>t\right]=E_{n}\left(X;t\right);$
**(ii)**  $\frac{1}{n}\;\mathrm{Cov}\left[X_{n},\Lambda \left(X_{n}\right)\;|\;X>t\right]=E_{n}\left(X;t\right).$

**Proof.**  (i) Recalling (33) we have

$$\begin{array}{ccc} \frac{1}{n}\;E\left[\frac{\Lambda \left(X_{n}\right)-\Lambda \left(t\right)}{\lambda (X_{n})}|X>t\right] & = & \frac{1}{n}\int_{t}^{\infty }\frac{\Lambda (x)-\Lambda (t)}{\lambda (x)}f_{n}\left(x\;|\;t\right)\;dt\\ \; & = & \int_{t}^{\infty }\frac{{\left[\Lambda \left(x\right)-\Lambda \left(t\right)\right]}^{n}}{n!}\frac{\bar{F}\left(x\right)}{\bar{F}\left(t\right)}\;dx, \end{array}$$

this giving the desired result due to the definition of DGCRE given in (v) of Table 1.

(ii) By the definition, we have

$$\mathrm{Cov}\left[X_{n},\Lambda \left(X_{n}\right)\;|\;X>t\right]=E\left[X_{n}\;\Lambda \left(X_{n}\right)|X>t\right]-E\left[X_{n}|X>t\right]\;E\left[\Lambda \left(X_{n}\right)|X>t\right].$$

From (33), one can easily obtain

$$E\left[X_{n}\;\Lambda \left(X_{n}\right)|X>t\right]=\int_{t}^{\infty }x\;\Lambda \left(x\right)\;f_{n}\left(x\;|\;t\right)\;dx=n\;E\left[X_{n+1}\;|\;X>t\right]+\Lambda \left(t\right)\;E\left[X_{n}\;|\;X>t\right]$$

and

$$E\left[\Lambda \left(X_{n}\right)\;|\;X>t\right]=\int_{t}^{\infty }\Lambda \left(x\right)\;f_{n}\left(x\;|\;t\right)\;dx=n+\Lambda \left(t\right),$$

so that

$$\mathrm{Cov}\left[X_{n},\Lambda \left(X_{n}\right)\;|\;X>t\right]=n\;\left(E\left[X_{n+1}\;|\;X>t\right]-E\left[X_{n}\;|\;X>t\right]\right).$$

Therefore, the result follows by means of (32). □

For t=0, Theorem 11 immediately yields the following result.

**Corollary** **1.** *For all n∈N it holds that*

**(i)**  $\frac{1}{n}\;E\left[\frac{\Lambda (X_{n})}{\lambda (X_{n})}\right]=E_{n}\left(X\right);$
**(ii)**  $\frac{1}{n}\;\mathrm{Cov}\left(X_{n},\Lambda \left(X_{n}\right)\right)=E_{n}\left(X\right).$

We remark also that $\Lambda \left(X_{n}\right)∼\mathrm{Gamma}\left(n,1\right)$, and thus $E[\Lambda \left(X_{n}\right)]=n.$ Moreover, the special case n=1 of Corollary 1 yields the relation Cov(X,Λ(X))=E(X), which is given in, e.g., [9].

Let us now provide a result for the variance residual life functions of two random variables satisfying the proportional mean residual life model (see Zahedi [34] and Nanda et al. [35] for some contributions on this topic).

**Theorem** **12.** *Let X and Y be absolutely continuous nonnegative random variables with survival functions $\bar{F}\left(t\right)$ and $\bar{G}\left(t\right),$ hazard functions $\lambda_{X}\left(t\right)$ and $\lambda_{Y}\left(t\right)$, and residual variances $\sigma^{2}\left(X_{t}\right)$ and $\sigma^{2}\left(Y_{t}\right),$ respectively. Assume that X and Y satisfy the proportional mean residual life model, so that*

(35) $$m_{Y}\left(t\right)=c\;m_{X}\left(t\right),\;\forall t>0,$$

*where c is constant. If c>1 and if $\sigma^{2}\left(X_{t}\right)$ is an increasing function of t, then $\sigma^{2}\left(Y_{t}\right)$ is also increasing in t, provided that $\lim_{t\to \infty }\frac{\bar{G}\left(t\right)}{\bar{F}\left(t\right)}<\infty .$*

**Proof.**  From differentiation of (35), after some calculations and making use of Equations (2) and (4), we first obtain that the hazard functions are related to the mean residual life of *X* by the following relation:

(36) $$\lambda_{X}\left(t\right)-\lambda_{Y}\left(t\right)=\frac{c-1}{c\;m_{X}\left(t\right)}.$$

Since $\sigma^{2}\left(X_{t}\right)$ is increasing in *t* by assumption, from (29) we have

(37) $$\sigma^{2}\left(X_{t}\right)\geq m_{X}^{2}\left(t\right)$$

for all t>0. We need to show that

$$\sigma^{2}\left(Y_{t}\right)\geq m_{Y}^{2}\left(t\right)=c^{2}m_{X}^{2}\left(t\right)$$

or, due to (37), the stronger condition

$$\frac{1}{c^{2}}\sigma^{2}\left(Y_{t}\right)\geq \sigma^{2}\left(X_{t}\right).$$

To that aim, we define the function

$$\varphi \left(t\right):=\bar{G}\left(t\right)\left[\sigma^{2}\left(X_{t}\right)-\frac{1}{c^{2}}\sigma^{2}\left(Y_{t}\right)\right].$$

We shall prove that φ(t)≤0. Differentiating φ(t) and using (29), (35), and (36), we have

$$\begin{array}{ccc} \varphi^{\prime }\left(t\right)\;\;\;\; & = & -g\left(t\right)\left[\sigma^{2}\left(X_{t}\right)-\frac{1}{c^{2}}\sigma^{2}\left(Y_{t}\right)\right]+\bar{G}\left(t\right)\left[\lambda_{X}\left(t\right)\left[\sigma^{2}\left(X_{t}\right)-m_{X}^{2}\left(t\right)\right]-\frac{1}{c^{2}}\lambda_{Y}\left(t\right)\left[\sigma^{2}\left(Y_{t}\right)-m_{Y}^{2}\left(t\right)\right]\right]\\ \; & = & \bar{G}\left(t\right)\left[\lambda_{X}\left(t\right)-\lambda_{Y}\left(t\right)\right]\left[\sigma^{2}\left(X_{t}\right)-m_{X}^{2}\left(t\right)\right]=\bar{G}\left(t\right)\frac{c-1}{c\;m_{X}\left(t\right)}\left[\sigma^{2}\left(X_{t}\right)-m_{X}^{2}\left(t\right)\right]. \end{array}$$

Hence, due to (37), since c>1, we have $\varphi^{\prime }\left(t\right)\geq 0$. From the fact that $\sigma^{2}\left(X_{t}\right)\geq 0$ and assumption $\lim_{t\to \infty }\frac{\bar{G}\left(t\right)}{\bar{F}\left(t\right)}<\infty ,$ recalling (28) we get

$$\lim_{t\to \infty }\varphi \left(t\right)=\lim_{t\to \infty }\bar{G}\left(t\right)\;\sigma^{2}\left(X_{t}\right)-\frac{1}{c^{2}}\lim_{t\to \infty }\bar{G}\left(t\right)\;\sigma^{2}\left(Y_{t}\right)=0.$$

Since φ(t) is increasing, this implies that φ(t)≤0, and the desired result then follows. □

The next theorem generalizes Theorem 4.5 of Asadi and Zohrevand [6] for the GCRE and the variance residual life function. We first recall the following result, which is stated in Navarro and Psarrakos [12].

**Lemma** **1.** *If X is IMRL (DMRL), then $E_{n}\left(X;t\right)$ is increasing (decreasing) in t>0, for all n∈N.*

**Theorem** **13.** *Let X and Y be absolutely continuous nonnegative random variables with survival functions $\bar{F}\left(t\right)$ and $\bar{G}\left(t\right),$ variance residual life functions $\sigma^{2}\left(X_{t}\right)$ and $\sigma^{2}\left(Y_{t}\right)$, and DGCRE functions $E_{n}\left(X;t\right)$ and $E_{n}\left(Y;t\right),$ respectively, such that $E_{n}\left(X;0\right)<\infty$ and $E_{n}\left(Y;0\right)<\infty ,$ for fixed n∈N. Let $X\leq_{hr}Y$, and let either X or Y be IMRL. Then*

 **(i)**  *$E_{n}\left(X;t\right)\leq E_{n}\left(Y;t\right),$ for all t>0 and for all n∈N;*
 **(ii)**  *$\sigma^{2}\left(X_{t}\right)\leq \sigma^{2}\left(Y_{t}\right),$ for all t>0.*

**Proof.**  (i) We prove it by induction, when *X* is IMRL. Assumption $X\leq_{hr}Y$ gives $m_{X}\left(t\right)\leq m_{Y}\left(t\right),$ for all t>0. Moreover, $X\leq_{hr}Y$ implies that E[ψ(X)|X>t]≤E[ψ(Y)|Y>t] for all increasing functions ψ(·), and in particular for $m_{X}\left(\cdot \right)$ since *X* is IMRL. Hence, from these conditions, recalling (31) one can obtain, for all t>0,

$$E_{1}\left(X;t\right)=\frac{\int_{t}^{\infty }m_{X}\left(x\right)\;f\left(x\right)\;dx}{\bar{F}\left(t\right)}\leq \frac{\int_{t}^{\infty }m_{X}\left(x\right)\;g\left(x\right)\;dx}{\bar{G}\left(t\right)}\leq \frac{\int_{t}^{\infty }m_{Y}\left(x\right)\;g\left(x\right)\;dx}{\bar{G}\left(t\right)}=E_{1}\left(Y;t\right).$$

Assume now that $E_{n-1}\left(X;t\right)\leq E_{n-1}\left(Y;t\right),$ for all t>0, and for a fixed n≥2. We recall that if *X* is IMRL, then $E_{n}\left(Y;t\right)$ is increasing in t>0, for all n∈N (see Lemma 1). Thus, using similar arguments of n=1, from (31) we obtain, for all t>0,

$$\begin{array}{ccc} E_{n}\left(X;t\right) & = & \frac{\int_{t}^{\infty }E_{n-1}\left(X;x\right)\;f\left(x\right)\;dx}{\bar{F}\left(t\right)}\leq \frac{\int_{t}^{\infty }E_{n-1}\left(X;x\right)\;g\left(x\right)\;dx}{\bar{G}\left(t\right)}\\ \; & \leq & \frac{\int_{t}^{\infty }E_{n-1}\left(Y;x\right)\;g\left(x\right)\;dx}{\bar{G}\left(t\right)}=E_{n}\left(Y;t\right), \end{array}$$

for n≥2. Thus, in this case the proof is completed. The case when *Y* is IMRL can be treated similarly.

(ii) By using similar arguments of part (i), and due to (28), the result is obtained. □

In the sequel, we compare the GCRE and variance residual life functions of two random variables under weaker conditions.

**Theorem** **14.** *Let X and Y be absolutely continuous nonnegative random variables with survival functions $\bar{F}\left(t\right)$ and $\bar{G}\left(t\right),$ mean residual life functions $m_{X}\left(t\right)$ and $m_{Y}\left(t\right)$, and DCRE functions $E_{n}\left(X;t\right)$ and $E_{n}\left(Y;t\right),$ respectively. If $X\leq_{mrl}Y$, and if X is DMRL and Y is IMRL, then*

**(i)**  *$E_{n}\left(X;t\right)\leq E_{n}\left(Y;t\right)$, for all t>0 and for all n∈N.*
**(ii)**  *$\sigma^{2}\left(X_{t}\right)\leq \sigma^{2}\left(Y_{t}\right)$, for all t>0.*

**Proof.**  (i) The proof proceeds by induction. For n=1, we have

(38) $$\begin{array}{ccc} E_{1}\left(Y;t\right)-E_{1}\left(X;t\right) & = & \frac{1}{\bar{G}\left(t\right)}\int_{t}^{\infty }m_{Y}\left(x\right)\;g\left(x\right)\;dx-\frac{1}{\bar{F}\left(t\right)}\int_{t}^{\infty }m_{X}\left(x\right)\;f\left(x\right)\;dx\\ \; & \geq & \frac{m_{Y}\left(t\right)}{\bar{G}\left(t\right)}\int_{t}^{\infty }g\left(x\right)\;dx-\frac{m_{X}\left(t\right)}{\bar{F}\left(t\right)}\int_{t}^{\infty }f\left(x\right)\;dx\\ \; & = & m_{Y}\left(t\right)-m_{X}\left(t\right)\geq 0. \end{array}$$

The first inequality in (38) is obtained by noting that *Y* is IMRL and *X* is DMRL, while the last inequality is obtained from the assumption $X\leq_{mrl}Y$. This gives the stated result for n=1. Now we assume that $E_{n-1}\left(X;t\right)\leq E_{n-1}\left(Y;t\right),$ for all t>0, and for a given n≥2. Since *X* is DMRL and *Y* is IMRL, from Lemma 1 we have that $E_{n-1}\left(X;t\right)$ and $E_{n-1}\left(Y;t\right)$ are decreasing and increasing, respectively. Hence, recalling (31) we get

$$\begin{array}{ccc} E_{n}\left(Y;t\right)-E_{n}\left(X;t\right) & = & \frac{1}{\bar{G}\left(t\right)}\int_{t}^{\infty }E_{n-1}\left(Y;t\right)\;g\left(x\right)\;dx-\frac{1}{\bar{F}\left(t\right)}\int_{t}^{\infty }E_{n-1}\left(X;t\right)\;f\left(x\right)\;dx\\ \; & \geq & \frac{E_{n-1}\left(Y;t\right)}{\bar{G}\left(t\right)}\int_{t}^{\infty }\;g\left(x\right)\;dx-\frac{E_{n-1}\left(X;t\right)}{\bar{F}\left(t\right)}\int_{t}^{\infty }\;f\left(x\right)\;dx\\ \; & = & E_{n-1}\left(Y;t\right)-E_{n-1}\left(X;t\right)\geq 0, \end{array}$$

where the last inequality is due to the inductive hypothesis. This completes the proof.

(ii) The proof of $\sigma^{2}\left(X_{t}\right)\leq \sigma^{2}\left(Y_{t}\right)$, t>0, can be obtained similarly, starting from (28), making use of the assumption that $m_{X}\left(x\right)$ and $m_{Y}\left(x\right)$ are decreasing and increasing, respectively, and finally, using $X\leq_{mrl}Y$. □

Finally, we conclude this section with a characterization of the exponential distribution in terms of variance residual life function and DCRE.

**Theorem** **15.** *Let X be a nonnegative, absolutely continuous random variable having support (0,∞), with survival function $\bar{F}\left(t\right)$, residual variance $\sigma^{2}\left(X_{t}\right)$, and DCRE E(X;t). Then*

(39) $$E\left(X;t\right)=\sigma \left(X_{t}\right)\;\forall t>0$$

*if and only if X is exponentially distributed.*

**Proof.**  The necessity is obvious. To prove the sufficiency, let Equation (39) hold so that

(40) $$E^{2}\left(X;t\right)=\sigma^{2}\left(X_{t}\right)\;\forall t>0.$$

By differentiating both sides of such identity with respect to t, recalling (29) and (30) for n=1, and (8), we obtain

$$2\lambda \left(t\right)\left[E^{2}\left(X;t\right)-m\left(t\right)E\left(X;t\right)\right]=\lambda \left(t\right)\left[\sigma^{2}\left(X_{t}\right)-m^{2}\left(t\right)\right].$$

Hence, from (40) we get

$$2E^{2}\left(X;t\right)-2m\left(t\right)E\left(X;t\right)=E^{2}\left(X;t\right)-m^{2}\left(t\right).$$

This reduces to ${\left[E\left(X;t\right)-m\left(t\right)\right]}^{2}=0$, which yields

E(X;t)=m(t),t>0.

The proof is thus complete; recal that the latter identity is fulfilled if and only if *X* is exponentially distributed (see Theorem 4.8 of Asadi and Zohrevand [6]). □

## 5. Applications in Reliability Theory

In the study of reliability of redundant systems, parallel systems play an important role. A parallel system consisting of *m* components, is a system which functions if and only if at least one of its *m* components functions. Let $X,X_{1},X_{2},\ldots ,X_{m}$ be i.i.d. random variables with common absolutely continuous distribution function *F* and survival function $\bar{F}=1-F,$ and assume that $X_{i}$, i=1,2,…,m, is the time up to the failure of the *i*th component of a coherent system. We denote by $X_{k:m}$, k=1,2,…,m, the lifetime of the component having the *k*th smallest lifetime among *m* i.i.d. system components. The random lifetime $X_{k:m}$ may represent the lifetime of an (m−k+1)-out-of-*m* system, which consists of *m* components and functions if and only if at least (m−k+1) out of *m* components functions. For the analysis of an (m−k+1)-out-of-*m* system, we can define $X_{r,k,m}\left(t\right)=\left[X_{k:m}-t|X_{r:m}>t\right],$ for all 1≤r≤k≤m. The conditional random variable $X_{r,k,m}\left(t\right)$ denotes the residual lifetime of the system under the condition that at least m−r+1 components of the system are working at time *t*. The survival function of this lifetime is expressed as

(41) F¯r,k,m(x|t)=∑i=0r−1miFi(t)F¯m−i(t)∑u=0k−i−1mm−iF¯(x)F¯(t)m−i−u1−F¯(x)F¯(t)u∑i=0r−1miFi(t)F¯m−i(t),

for all x≥t>0. We denote the corresponding mean residual lifetime by

$$m_{r,k,m}\left(X,t\right)=E\left[X_{k:m}-t|X_{r:m}>t\right],\;1\leq r\leq k\leq m.$$

Asadi and Goliforushani [36] provided the following expression

(42) $$m_{r,k,m}\left(X,t\right)=\sum_{i=0}^{r-1}p_{i}\left(t\right)m_{1,k-i,m-i}\left(X,t\right),$$

where

pi(t)=miϕi(t)∑j=0r−1mjϕj(t),

and where $\phi \left(t\right)=F\left(t\right)/\bar{F}\left(t\right),\;t>0,$ is the odds function. This measure has been studied by several authors in the reliability literature. Applying (42) and recalling (12), the residual variance of $X_{r,k,m}\left(t\right)$, for t>0 will be denoted

(43) $$\sigma_{r,k,m}^{2}\left(X,t\right)=V\mathrm{ar}\left[X_{k:m}-t|X_{r:m}>t\right]=\int_{t}^{\infty }m_{r,k,m}^{2}\left(X,x\right)f_{r,k,m}\left(x|t\right)\;dx$$

for all 1≤r≤k≤m, where

$$f_{r,k,m}\left(x|t\right)=-\frac{d}{dx}{\bar{F}}_{r,k,m}\left(x|t\right)$$

is the pdf of $X_{r,k,m}\left(t\right)$. Recalling (31), one can obtain the GCRE of $X_{r,k,m}\left(t\right)$ as

$$E_{n}\left(X_{r,k,m}\left(t\right)\right)=\int_{t}^{\infty }E_{n-1}\left(X_{r,k,m}\left(x\right)\right)f_{r,k,m}\left(x|t\right)\;dx,\;t>0,$$

for all 1≤r≤k≤m, and for all n∈N.

Asadi and Goliforushani [36] have shown that, when the components of an (m−k+1)-out-of-*m* system are IFR, then the mean residual life function of $X_{r,k,m}\left(t\right)$ is a decreasing function of time *t*. In the following theorem, we prove similar results for the variance residual life function and the GCRE. We just prove it for the variance since the proof for the other measure is similar.

**Theorem** **16.** *Let $X_{1},\ldots ,X_{n}$ denote the i.i.d. lifetimes of an (m−k+1)-out-of-m system. If X is IFR, then $\sigma_{r,k,m}^{2}\left(X,t\right)$ and $E_{n}\left(X_{r,k,m}\left(t\right)\right)$ are decreasing functions of t.*

**Proof.**  The results simply follow from Theorem 2.4 of Asadi and Goliforushani [36], Theorem 3.3 of Gupta [32] and making use of Lemma 1. □

Other comparisons of (m−k+1)-out-of-*m* systems are given in the next two theorems.

**Theorem** **17.** *Let $X_{1},\ldots ,X_{n}$ denote the lifetimes of an (m−k+1)-out-of-m system. If X is DFR, then*

**(i)**  *$\sigma_{1,k-1,m-1}^{2}\left(X,t\right)\leq \sigma_{1,k,m}^{2}\left(X,t\right)$ for all t>0;*
**(ii)**  *$E_{n}\left(X_{1,k-1,m-1}\left(t\right)\right)\leq E_{n}\left(X_{1,k,m}\left(t\right)\right)$ for all t>0.*

**Proof.**  (i) Let *X* be DFR. Then, from (43) we have

$$\begin{array}{ccc} \sigma_{1,k-1,m-1}^{2}\left(X,t\right) & = & \int_{t}^{\infty }m_{1,k-1,m-1}^{2}\left(X,x\right)f_{1,k-1,m-1}\left(x|t\right)\;dx\\ \; & \leq & \int_{t}^{\infty }m_{1,k,m}^{2}\left(X,x\right)f_{1,k-1,m-1}\left(x|t\right)\;dx\\ \; & \leq & \int_{t}^{\infty }m_{1,k,m}^{2}\left(X,x\right)f_{1,k,m}\left(x|t\right)\;dx=\sigma_{1,k,m}^{2}\left(X,t\right). \end{array}$$

The first inequality is obtained due to Remark 3 of Asadi and Bayramoglu [37] by noting that $m_{1,k-1,m-1}^{2}\left(X,x\right)\leq m_{1,k,m}^{2}\left(X,x\right)$ for all x>0, while the second inequality follows by the fact that when *X* is DFR, then $m_{1,k,m}\left(X,x\right)$ is an increasing function of *x* due to Theorem 2 of Asadi and Bayramoglu [37] and by noting that $X_{1,k-1,m-1}\left(t\right)\leq_{lr}X_{1,k,m}\left(t\right)$ which implies $X_{1,k-1,m-1}\left(t\right)\leq_{hr}X_{1,k,m}\left(t\right)$ for all t>0. The latter relation can be obtained from the following remarks: from Remark 1 of Asadi and Bayramoglu [37], we derive that the distribution of $X_{1,k,m}\left(t\right)$ is identical to the distribution of the *k*-th order statistics of the sample taken from the conditional distribution of [X−t|X>t]. Therefore, from Corollary 1.C.38 of Shaked and Shanthikumar [26] it holds that $X_{1,k-1,m-1}\left(t\right)\leq_{lr}X_{1,k,m}\left(t\right)$, which implies $X_{1,k-1,m-1}\left(t\right)\leq_{hr}X_{1,k,m}\left(t\right)$ for all t>0.

(ii) The proof is similar to that of (i) and thus is omitted. □

The following theorem gives sufficient conditions for comparison of two (m−k+1)-out-of-*m* systems based on their variance residual life functions and dynamic GCREs.

**Theorem** **18.** *Let us consider two (m−k+1)-out-of-m systems with i.i.d. component lifetimes distributed as X and Y, and having distribution functions F and G, respectively. If $X\leq_{hr}Y$ and X is DFR, then*

 **(i)**  *$\sigma_{1,k,m}^{2}\left(X,t\right)\leq \sigma_{1,k,m}^{2}\left(Y,t\right)$ for all t>0;*
 **(ii)**  *$E_{n}\left(X_{1,k,m}\left(t\right)\right)\leq E_{n}\left(Y_{1,k,m}\left(t\right)\right)$ for all t>0.*

**Proof.**  For t>0, from (43) we have

$$\begin{array}{ccc} \sigma_{1,k,m}^{2}\left(X,t\right) & = & \int_{t}^{\infty }m_{1,k,m}^{2}\left(X,t\right)f_{1,k,m}\left(x|t\right)\;dx\\ \; & \leq & \int_{t}^{\infty }m_{1,k,m}^{2}\left(X,t\right)g_{1,k,m}\left(x|t\right)\;dx\\ \; & \leq & \int_{t}^{\infty }m_{1,k,m}^{2}\left(Y,t\right)g_{1,k,m}\left(x|t\right)\;dx=\sigma_{1,k,m}^{2}\left(Y,t\right). \end{array}$$

Since *X* is DFR, then $m_{1,k,m}^{2}\left(X,t\right)$ is an increasing function of *t* due to Theorem 2 of Asadi and Bayramoglu [37]. Moreover, $X\leq_{hr}Y$ implies $X_{1,k,m}\left(t\right)\leq_{hr}Y_{1,k,m}\left(t\right)$ and hence the first inequality is obtained. The second inequality follows from Theorem 3 of Asadi and Bayramoglu [37]. Finally, the proof of part (ii) is similar to the proof of part (i) and thus is omitted. □

## 6. Results on the GCE

As pointed out by Kayal [16], there is an intimate connection with GCE and lower record values. Let $Y_{1},Y_{2},\ldots$ be a sequence of i.i.d. nonnegative random variables with a common absolutely continuous cdf *F*, and with pdf f(x). An observation $Y_{i}$ is said to be a lower record if $Y_{i}<Y_{j}$ for all j<i. Assume that if $Y_{i}$ occurs at time i, then the record time sequence is defined as

$$L\left(1\right)=1,\;L\left(n+1\right)=\min\left\{i:Y_{i}<Y_{L(n)}\right\},\;n\in N.$$

The random variables $X_{n+1}^{🟉}=Y_{L(n+1)}$, $n\in N_{0}$, are said to be the lower records, such that $Y_{L(1)}\overset{d}{=}X.$ Denoting by $F_{n+1}^{🟉}\left(x\right)$ the cumulative distribution function of $X_{n+1}^{🟉},$$n\in N_{0},$ it follows that

(44) $$F_{n+1}^{🟉}\left(x\right)=F\left(x\right)\;\sum_{k=0}^{n}\frac{T^{k}\left(x\right)}{k!},\;x\geq 0,$$

so that the pdf of $X_{n+1}^{🟉}$ is given by

(45) $$f_{n+1}^{🟉}\left(x\right)=f\left(x\right)\frac{T^{n}\left(x\right)}{n!},\;x\geq 0,$$

where T(x) is the cumulative reversed hazard function defined in (5). Recalling the definition of GCE given in case (iv) of Table 1, from (44) we obtain

(46) $$\begin{array}{ccc} {\mathrm{CE}}_{n}\left(X\right) & = & E\left[X_{n}^{🟉}-X_{n+1}^{🟉}\right]=\int_{0}^{\infty }\left[F_{n+1}^{🟉}\left(x\right)-F_{n}^{🟉}\left(x\right)\right]dx,\\ \; & = & \int_{0}^{\infty }\frac{T^{n}\left(x\right)}{n!}F\left(x\right)\;dx,\;n\in N. \end{array}$$

Thus, the GCE of order *n* corresponds to the expected spacings of lower record values. Note that for $n=0,\;{\mathrm{CE}}_{0}\left(X\right)=\int_{0}^{\infty }F\left(x\right)dx,$ which may be divergent. With reference to the GCE, in the following theorem we obtain a result analogous to (14). The proof is omitted, being similar to that of Theorem 3 since

$$\int_{t}^{\infty }\frac{T^{n-1}\left(x\right)}{(n-1)!}\;\tau \left(x\right)\;dx=\frac{T^{n}\left(t\right)}{n!},\;t\geq 0,\;\;n\in N.$$

**Theorem** **19.** *Let X be an absolutely continuous nonnegative random variable with mean inactivity time function $\tilde{\mu }\left(t\right)$. Then, for all n∈N one has*

$${\mathrm{CE}}_{n}\left(X\right)=E\left[\tilde{\mu }\left(X_{n}^{🟉}\right)\right].$$

In the following theorem, we determine equivalent expressions for the GCE analogous to those given in Theorems 4 and 5, and thus the proof is omitted.

**Theorem** **20.** *Under the assumption of Theorem 19, for all n∈N, we have*

**(i)**  $${\mathrm{CE}}_{n}\left(X\right)={\mathrm{CE}}_{n-1}\left(X\right)-\frac{1}{(n-1)!}\;E\left[{\tilde{h}}_{n}\left(X\right)\right],$$
  *where*
  $${\tilde{h}}_{n}\left(t\right):=\int_{t}^{\infty }{\tilde{\mu }}^{\prime }\left(x\right)\;T^{n-1}\left(x\right)\;dx.$$
**(ii)**  $${\mathrm{CE}}_{n}\left(X\right)={\mathrm{CE}}_{n-1}\left(X\right)\left\{1-E[{\tilde{\mu }}^{\prime }\left(\tilde{Z}\right)]\right\},$$
  *where $\tilde{Z}$ is an absolutely continuous nonnegative random variable having pdf*
  $$f_{\tilde{Z}}\left(x\right)=\frac{F(x)}{{\mathrm{CE}}_{n-1}\left(X\right)}\;\frac{T^{n-1}\left(x\right)}{(n-1)!},\;x>0.$$

For an absolutely continuous nonnegative random variable X, for all t>0 such that F(t)>0, the mean inactivity time (6) can be written as

(47) $$\tilde{\mu }\left(t\right)=E\left[t-X|X\leq t\right]=t-\mu \left(t\right),$$

where

$$\mu \left(t\right)=E\left[X|X\leq t\right]=\frac{1}{F(t)}\int_{0}^{t}xf\left(x\right)\;dx$$

denotes the mean failure time of a system conditioned by a failure before time t, also named “mean past lifetime”. The derivative of this function is given by

(48) $$\mu^{\prime }\left(t\right)=t\tau \left(t\right)-\tau \left(t\right)\mu \left(t\right),$$

for all t>0. In the next theorem, we prove that the variance of a random variable can also be written in terms of the mean inactivity time function.

**Theorem** **21.** *Let X be an absolutely continuous nonnegative random variable with pdf f, cdf F, and mean inactivity time $\tilde{\mu }\left(x\right),$ where the second moment $E(X^{2})$ is finite. Then*

(49) $$\sigma^{2}\left(X\right)=E\left[{\tilde{\mu }}^{2}\left(X\right)\right].$$

**Proof.**  Let us set

$$w\left(x\right):=\mu \left(x\right)F\left(x\right)=\int_{0}^{x}tf\left(t\right)\;dt,\;x>0.$$

Using (47), we obtain

(50) $$E\left[{\tilde{\mu }}^{2}\left(X\right)\right]=\int_{0}^{\infty }{\left[x-\mu \left(x\right)\right]}^{2}f\left(x\right)\;dx=E\left(X^{2}\right)+\int_{0}^{\infty }\mu^{2}\left(x\right)f\left(x\right)\;dx-2\int_{0}^{\infty }x\mu \left(x\right)f\left(x\right)\;dx.$$

Recalling (48), it holds that

$$\begin{array}{ccc} \int_{0}^{\infty }\mu^{2}\left(x\right)f\left(x\right)\;dx & = & \int_{0}^{\infty }\mu \left(x\right)\tau \left(x\right)w\left(x\right)\;dx=\int_{0}^{\infty }x\tau \left(x\right)w\left(x\right)\;dx-\int_{0}^{\infty }\mu^{\prime }\left(x\right)w\left(x\right)\;dx\\ \; & = & \int_{0}^{\infty }x\mu \left(x\right)f\left(x\right)\;dx-\int_{0}^{\infty }\mu^{\prime }\left(x\right)w\left(x\right)\;dx. \end{array}$$

Integration by parts gives

$$\int_{0}^{\infty }\mu^{\prime }\left(x\right)w\left(x\right)\;dx={\left[E\left(X\right)\right]}^{2}-\int_{0}^{\infty }x\mu \left(x\right)f\left(x\right)\;dx,$$

which implies

(51) $$\begin{array}{c} \int_{0}^{\infty }\mu^{2}\left(x\right)f\left(x\right)\;dx=2\int_{0}^{\infty }x\mu \left(x\right)f\left(x\right)\;dx-{\left[E\left(X\right)\right]}^{2}. \end{array}$$

By substituting Equation (51) into (50), we have

$$E\left[{\tilde{\mu }}^{2}\left(X\right)\right]=E\left(X^{2}\right)-{\left[E\left(X\right)\right]}^{2}\equiv \sigma^{2}\left(X\right),$$

and this gives (49). □

In analogy with the result given in Theorem 2, on the ground of identity (49) we can now state the following theorem. First we remark that for two absolutely continuous nonnegative random variables *X* and *Y* with cdf’s *F* and *G*, pdf’s *f* and g, and mean inactivity time functions ${\tilde{\mu }}_{X}\left(t\right)$ and ${\tilde{\mu }}_{Y}\left(t\right),$ respectively, we say that *X* is smaller than *Y* in the mean inactivity time order, denoted by $X\leq_{\mathrm{mit}}Y,$ if ${\tilde{\mu }}_{X}\left(t\right)\geq {\tilde{\mu }}_{Y}\left(t\right)$ for all t>0. Moreover, we recall that *X* is said to have increasing mean inactivity time (IMIT) if $\tilde{\mu }\left(t\right)$ is increasing in t>0. The proof of the following theorem is omitted, being similar to that of Theorem 2.

**Theorem** **22.** *Let X and Y be nonnegative random variables with mean inactivity time functions ${\tilde{\mu }}_{X}\left(t\right)$ and ${\tilde{\mu }}_{Y}\left(t\right),$ respectively. Let $X\leq_{st}Y$ and $X\leq_{mit}Y$. If either X or Y is IMIT, then $\sigma^{2}\left(X\right)\leq \sigma^{2}\left(Y\right).$*

Analogously to (19), hereafter we obtain an upper bound for the GCE in terms of the expected value of the squared mean inactivity time. The proof is omitted being similar to that of Theorem 6.

**Theorem** **23.** *Let X be an absolutely continuous nonnegative random variable with mean inactivity time $\tilde{\mu }\left(t\right)$. Then, for all n∈N,*

$${\mathrm{CE}}_{n}\left(X\right)\leq \frac{\sqrt{[2(n-1)]!}}{(n-1)!}\;\sigma \left(X\right).$$

In the special case n=1, from Theorem 23, one can obtain a close relationship between the standard deviation and the cumulative entropy; i.e.,

$$e^{CH(X)}\leq {\mathrm{CE}}_{1}\left(X\right)\leq \sigma \left(X\right),$$

similarly as in (21).

## 7. Results on Past Variance and DGCE

In analogy with the variance of the residual life function defined in (27), we can introduce the variance of inactivity time (VIT) as (see, e.g., Mahdy [38])

(52) $$\begin{array}{ccc} \sigma^{2}\left[X_{\left[t\right]}\right] & := & V\mathrm{ar}\left[t-X\;|\;X\leq t\right]=E\left[{\left(t-X\right)}^{2}|X\leq t\right]-{\tilde{\mu }}^{2}\left(t\right)\\ \; & = & \frac{2}{F(t)}\int_{0}^{t}\int_{0}^{x}F\left(u\right)\;du\;dx-{\tilde{\mu }}^{2}\left(t\right),\;t>0. \end{array}$$

It should be noted that it is of interest to compare the dispersion of inactivity time distributions in some situations, especially when the mean inactivity times of these distributions are non-ordered. For example, suppose that the random variable *X* represents the failure time (or time of death) of a component or a living organism. Assume that the a component or a patient with lifetime X, fails (dies) at time *t* or sometimes before *t*, and consequently the component can be considered as a black box in the sense that the exact failure time or drying time of *X* is unknown. In this case, one wishes to estimate the time that has elapsed since the failure and to study the dispersion of this elapsed interval of time. Therefore, several authors have considered properties and stochastic orders of the variance inactivity time function; see Mahdy [38,39] and Kayid and Izadkhah [40] and the references therein. Similarly as (49), one can obtain an analogue representation for the variance inactivity time function in terms of the mean inactivity time function as follows:

(53) $$\sigma^{2}\left[X_{\left[t\right]}\right]=E\left[{\tilde{\mu }}^{2}\left(X\right)|X\leq t\right],\;t>0.$$

In analogy with (46) we have:

$$\begin{array}{c} {\mathrm{CE}}_{n}\left(X;t\right)=E\left[X_{n}^{🟉}-X_{n+1}^{🟉}\;|\;X\leq t\right]=\int_{0}^{t}\frac{F(x)}{F(t)}\frac{{\left[T\left(x\right)-T\left(t\right)\right]}^{n}}{n!}\;dx,\;n\in N. \end{array}$$

Hereafter, we provide a result that is dual to Theorem 10, and hence its proof is omitted. To that end, we note that

(54) $$f_{n}^{🟉}\left(x\;|\;t\right):=\frac{{\left[T\left(x\right)-T\left(t\right)\right]}^{n-1}}{(n-1)!}\frac{f(x)}{F(t)},\;0<x\leq t,$$

is the pdf of $\left[X_{n}^{🟉}\;|\;X\leq t\right]$, n∈N.

**Theorem** **24.** *Under the assumptions of Theorem 19, for all n∈N and t≥0, we have*

$${\mathrm{CE}}_{n}\left(X;t\right)=E\left[\tilde{\mu }\left(X_{n}^{🟉}\right)\;|\;X\leq t\right].$$

We remark that the dynamic version of Theorem 23 provides the following upper bound:

$${\mathrm{CE}}_{n}\left(X;t\right)\leq \frac{\sqrt{[2(n-1)]!}}{(n-1)!}\;\sigma \left[X;t\right],\;n\in N,\;t\geq 0.$$

Now, with reference to Theorem 11, we obtain the dual results for the past time. In fact, through two suitable expressions we provide different probabilistic meanings for the DGCE where the first is given in terms of a conditional expectation involving the cumulative reversed hazard function as well as the reversed hazard rate function.

**Theorem** **25.** *For any t≥0 and for all n∈N, it holds that*

**(i)**  $\frac{1}{n}\;E\left[\frac{T\left(X_{n}^{🟉}\right)-T\left(t\right)}{\tau (X_{n}^{🟉})}\;|\;X\leq t\right]={\mathrm{CE}}_{n}\left(X;t\right);$
**(ii)**  $\frac{1}{n}\;\mathrm{Cov}\left[X_{n}^{🟉},T\left(X_{n}^{🟉}\right)\;|\;X\leq t\right]=-{\mathrm{CE}}_{n}\left(X;t\right).$

The analogies between Equations (27) and (52) allow us to provide various results for the VIT that are similar to those for the variance of the residual life. The following theorem is an analogue to Theorem 12; the proof is similar and thus is omitted. In this case we consider random variables having proportional mean inactivity time functions.

**Theorem** **26.** *Let X and Y be absolutely continuous nonnegative random variables with survival functions $\bar{F}\left(t\right)$ and $\bar{G}\left(t\right),$ mean inactivity time functions ${\tilde{\mu }}_{X}\left(t\right)$ and ${\tilde{\mu }}_{Y}\left(t\right)$, and variances of inactivity time $\sigma^{2}\left[X_{\left[t\right]}\right]$ and $\sigma^{2}\left[Y_{\left[t\right]}\right],$ respectively. Assume that*

(55) $${\tilde{\mu }}_{Y}\left(t\right)=c\;{\tilde{\mu }}_{X}\left(t\right),\;\forall t>0,$$

*where c is constant. If c>1 and if $\sigma^{2}\left[X_{\left[t\right]}\right]$ is an increasing function of t, then $\sigma^{2}\left[Y_{\left[t\right]}\right]$ is also increasing in t.*

We conclude with a result for the generalized cumulative entropy and the variance of inactivity time that is analogous to Theorem 13. Again, the proof is similar and thus is omitted. We have seen already that *X* is IMIT means that $\tilde{\mu }\left(t\right)$ is increasing in t>0. Moreover, consider the following lemma, which is stated in Di Crescenzo and Toomaj [17].

**Lemma** **2.** *If X is IMIT, then ${\mathrm{CE}}_{n}\left(X;t\right)$ is increasing in t>0, for all n∈N.*

**Theorem** **27.** *Let X and Y be absolutely continuous nonnegative random variables with variances of inactivity time $\sigma^{2}\left[X_{\left[t\right]}\right]$ and $\sigma^{2}\left[Y_{\left[t\right]}\right]$, and DGCE functions ${\mathrm{CE}}_{n}\left(X;t\right)$ and ${\mathrm{CE}}_{n}\left(Y;t\right),$ respectively, such that ${\mathrm{CE}}_{n}\left(X;0\right)<\infty ,$ and ${\mathrm{CE}}_{n}\left(Y;0\right)<\infty ,$ for fixed n∈N. Let $X\leq_{rhr}Y$, and let either X or Y be IMIT. Then*

**(i)**  *${\mathrm{CE}}_{n}\left(X;t\right)\leq {\mathrm{CE}}_{n}\left(Y;t\right),$ for all t>0 and for all n∈N;*
**(ii)**  *$\sigma^{2}\left[X_{\left[t\right]}\right]\leq \sigma^{2}\left[Y_{\left[t\right]}\right]$, for all t>0.*

## 8. Conclusions

Besides that the GCRE is related to the upper record values of a sequence of independent and identically distributed random variables, and the relevation transform, there is also an intimate connection of the GCRE with the non-homogeneous poisson process, so we are assured that several findings in the literature on the GCRE can be used in shock models, preventive maintenance, repairable systems, and standby systems. It is known that the GCRE has some important variability properties, such as location invariance and positive homogeneity (i.e., $E_{n}\left(aX+b\right)=aE_{n}\left(X\right)$ for all a>0 and b∈R) and standardization (i.e., for all c∈R, one has $E_{n}\left(c\right)=0$). These properties suggest that the GCRE can also be considered as a dispersion measure. In this case, a connection of the GCRE with the standard deviation is derived. We have shown that when a random variable is absolutely continuous, then its variance can be represented in terms of either mean residual lifetime and mean inactivity time. Based on these expressions, several preservation properties with some well-known stochastic orders have been investigated. Moreover, the connection of the GCRE with the excess wealth order has been studied, together with some properties of the dynamic version of GCRE. Similar results have been obtained also for the GCE, being the dual measure of GCRE.

Future developments based on the ideas considered in this paper will be oriented to the case of weighted measures involving cumulative weighted random variables (see Toomaj and Di Crescenzo [41]). Other related research lines may involve (i) the information-based measures of statistical dispersion based on entropy and Fisher information treated by Kostal et al. [42], and (ii) the variance of typical information measures, such as the differential entropy and the Tsallis entropy (see, e.g., Di Crescenzo and Paolillo [43], and Wei [44]).

Finally, we note that a new kind of entropy that combines the concepts of CRE and CE is the cumulative paired ϕ-entropy. The construction of such a notion is based on both the distribution function and the survival function of *X*, through the functional

$$CPE_{\phi }\left(X\right)=\int_{R}\left[\phi \left(F\left(x\right)\right)+\phi \left(\bar{F}\left(x\right)\right)\right]dx.$$

See Klein et al. [45] and Klein and Doll [46] for detailed investigations on $CPE_{\phi }$, also dealing with relationships of the cumulative paired entropy to the variance. This entropy has the properties of a measure of scale (or dispersion). Suitable choices of ϕ allow one to introduce suitable generalized measures. This approach can be adopted also to construct paired combinations of the CGRE and GCE, and of their dynamic counterparts. This reveals new research ideas for future developments.

## Figures and Tables

**Table 1:** Information measures of interest, for a given random lifetime *X*, with $n\in N_{0}$ for cases (iii) and (v), n∈N for cases (iv) and (vi), and t>0 for cases (v) and (vi).

(i) cumulative residual entropy (CRE)	(ii) cumulative entropy (CE)
$\begin{array}{lll} E\left(X\right) & = & -\int_{0}^{\infty }\bar{F}\left(x\right)\log\bar{F}\left(x\right)\;dx\\ & = & \int_{0}^{\infty }\bar{F}\left(x\right)\Lambda \left(x\right)\;dx \end{array}$	$\begin{array}{lll} \mathrm{CE}\left(X\right) & = & -\int_{0}^{\infty }F\left(x\right)\log F\left(x\right)\;dx\\ & = & \int_{0}^{\infty }F\left(x\right)T\left(x\right)\;dx \end{array}$
(iii) generalized cumulative residual entropy (GCRE)	(iv) generalized cumulative entropy (GCE)
$\begin{array}{c} E_{n}\left(X\right)=\frac{1}{n!}\int_{0}^{\infty }\bar{F}\left(x\right){\left[\Lambda \left(x\right)\right]}^{n}\;dx\\ =\frac{1}{n!}\int_{0}^{\infty }\bar{F}\left(x\right){\left[-\log\bar{F}\left(x\right)\right]}^{n}\;dx \end{array}$	$\begin{array}{c} {\mathrm{CE}}_{n}\left(X\right)=\frac{1}{n!}\int_{0}^{\infty }F\left(x\right)\;{\left[T\left(x\right)\right]}^{n}\;dx\\ =\frac{1}{n!}\int_{0}^{\infty }F\left(x\right){\left[-\log F\left(x\right)\right]}^{n}\;dx \end{array}$
(v) dynamic gen. cumulative residual entropy (DGCRE)	(vi) dynamic gen. cumulative entropy (DGCE)
$\begin{array}{c} E_{n}\left(X;t\right)=\frac{1}{n!}\int_{t}^{\infty }\frac{\bar{F}\left(x\right)}{\bar{F}\left(t\right)}{\left[-\log\frac{\bar{F}\left(x\right)}{\bar{F}\left(t\right)}\right]}^{n}dx\\ =\frac{1}{n!}\int_{t}^{\infty }\frac{\bar{F}\left(x\right)}{\bar{F}\left(t\right)}\;{\left[\Lambda \left(x\right)-\Lambda \left(t\right)\right]}^{n}\;dx \end{array}$	$\begin{array}{lll} {\mathrm{CE}}_{n}\left(X;t\right) & = & \frac{1}{n!}\int_{0}^{t}\frac{F(x)}{F(t)}{\left[-\log\frac{F(x)}{F(t)}\right]}^{n}dx\\ & = & \frac{1}{n!}\int_{0}^{t}\frac{F(x)}{F(t)}{\left[T(x)-T(t)\right]}^{n}dx \end{array}$
